# Gonadotropin-Releasing Hormone (GnRH) and Its Agonists in Bovine Reproduction I: Structure, Biosynthesis, Physiological Effects, and Its Role in Estrous Synchronization

**DOI:** 10.3390/ani14101473

**Published:** 2024-05-15

**Authors:** Eman M. Hassanein, Zoltán Szelényi, Ottó Szenci

**Affiliations:** 1 Department of Obstetrics and Food Animal Medicine Clinic, University of Veterinary Medicine Budapest, H-2225 Üllő, Hungary; em.mostafa@alexu.edu.eg (E.M.H.); szelenyi.zoltan@univet.hu (Z.S.); 2 Animal and Fish Production Department, Faculty of Agriculture, Alexandria University, Alexandria 21545, Egypt

**Keywords:** GnRH analog, gonadotropins, hypothalamic–pituitary axis, follicular dynamic, estrous synchronization, reproductive performance, dairy cattle

## Abstract

**Simple Summary:**

Gonadotropin-releasing hormone (GnRH), a critical regulator of pituitary gonadotropin secretion, plays a fundamental role in controlling physiological aspects of mammalian reproduction. GnRH is produced in the hypothalamus and regulates the release of luteinizing hormone (LH) and follicle-stimulating hormone (FSH) from the anterior pituitary. GnRH analogs, including agonists and antagonists, have been developed for the reproductive management of animals. This review focuses on the functions of hypothalamic GnRH in reproductive processes and the enhancement of reproductive efficiency in farm animals. Various GnRH analogs have been synthesized to improve their potency and reproductive function. This review specifically highlights the application of GnRH in estrus synchronization to increase the reproductive efficiency of dairy cows.

**Abstract:**

GnRH is essential for the regulation of mammalian reproductive processes. It regulates the production and release of pituitary gonadotropins, thereby influencing steroidogenesis and gametogenesis. While primarily produced in the hypothalamus, GnRH is also produced in peripheral organs, such as the gonads and placenta. GnRH analogs, including agonists and antagonists, have been synthesized for the reproductive management of animals and humans. This review focuses on the functions of hypothalamic GnRH in the reproductive processes of cattle. In addition to inducing the surge release of LH, the pulsatile secretion of GnRH stimulates the pituitary gland to release FSH and LH, thereby regulating gonadal function. Various GnRH-based products have been synthesized to increase their potency and efficacy in regulating reproductive functions. This review article describes the chemical structures of GnRH and its agonists. This discussion extends to the gene expression of GnRH in the hypothalamus, highlighting its pivotal role in regulating the reproductive process. Furthermore, GnRH is involved in regulating ovarian follicular development and luteal phase support, and estrus synchronization is involved. A comprehensive understanding of the role of GnRH and its analogs in the modulation of reproductive processes is essential for optimizing animal reproduction.

## 1. Introduction

**Figure 1:**
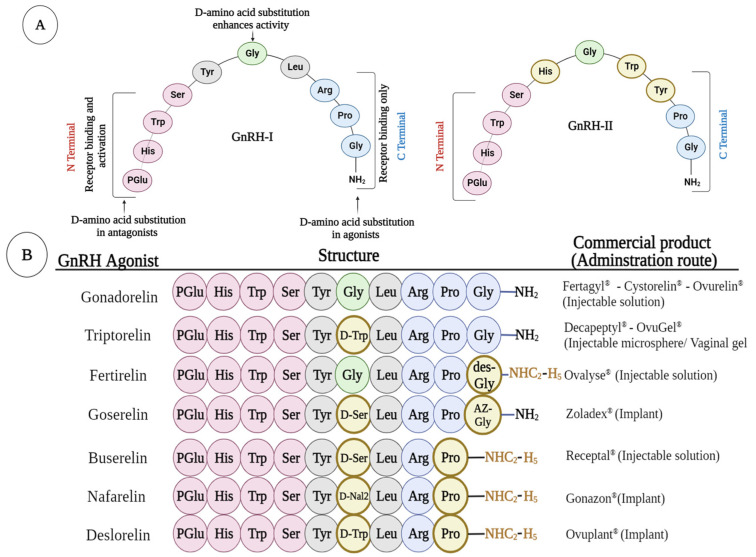
Structural depiction of (**A**) GnRH isoform (GnRH-I and GnRH-II) and (**B**) GnRH agonists, including their commercial products and administration route. Created with BioRender.com.

GnRH, formerly known as luteinizing hormone-releasing hormone (LHRH), is a decapeptide produced in the hypothalamus. It was initially identified as a neuropeptide that regulates secretions from the anterior pituitary gland [1]. While GnRH serves various functions in mammals, research has mainly focused on its role as a gonadotropin-releasing factor, influencing reproductive processes by regulating the production and release of pituitary gonadotropins, thereby affecting steroidogenesis and gametogenesis [2,3]. This essential function has sustained ongoing interest in GnRH because of its significance as a critical regulator of reproductive functions across species. GnRH is produced in the hypothalamus and peripheral organs such as the gonads and placenta, referred to as extrahypothalamic GnRH [1]. In vertebrates, three distinct types of GnRH are GnRH-I (mammalian GnRH), GnRH-II (chicken GnRH), as depicted in Figure 1, and GnRH-III (lamprey GnRH). Only GnRH I and GnRH II and their associated receptors have been found in mammals [4]. GnRH-I was the first structurally characterized isoform, whereas GnRH-II, isolated from the chicken brain, represents the second identified structure in vertebrates. Even though mammals produce both isoforms, only GnRH-I has physiological activities and functions, particularly in modulating reproductive functions [5,6]. GnRH-II appears predominantly expressed in the peripheral organs, where it is believed to act as an autocrine or paracrine agent, regulating ovarian processes and apoptosis in ovarian structures [7]. Thus, this review article focuses on mammalian hypothalamic GnRH.

In the hypothalamic–pituitary–gonadal (HPG) axis, pulsatile secretion of GnRH into the portal circulation regulates the biosynthesis and secretion of FSH and LH from the anterior pituitary gland. In adults, FSH and LH, known as gonadotropins, regulate the functions of the gonads (testes and ovaries) by stimulating gamete production and steroid hormone synthesis in both sexes [8]. Several factors are essential for regulating the pulsation of GnRH to maintain hormonal balance. These factors include feedback mechanisms, which can be either negative or positive, in response to changes in the concentration of gonadal steroid hormones during different phases of the estrous cycle in females. In addition, neurotransmitters, aging, and sex also have an impact on the release of GnRH pulsations [9]. In males, the pulsatile secretion of GnRH plays a vital role in controlling spermatogenesis and the release of gonadotropins, mainly LH. LH, in turn, induces Leydig cells to release testosterone, whereas FSH stimulates Sertoli cells to produce inhibin. Inhibin provides negative feedback to the HPG axis, inhibiting the secretion of FSH [10]. This sophisticated hormonal regulation occurs through the portal vasculature’s capillaries within the hypothalamic–pituitary tissues’ stalk median eminence [11].

Synthesis of GnRH-based products, including agonists and antagonists, commenced in the 1970s following the discovery of the mammalian GnRH structure in the early 1970s [12,13]. These products were developed to address reproductive dysfunctions and enhance efficiency in farm animals [14]. In dairy cows, the reproductive cycle is characterized by main phases: the follicular phase and the luteal phase. These phases involve unique temporary endocrine functions of the ovarian structures. Consequently, GnRH-based products are essential in breeding protocols to synchronize estrous stages and improve reproductive performance [15,16]. Although there are other methods available, GnRH is undoubtedly the best option. This review highlights the structure of GnRH and its agonists, showing the diverse functions of GnRH, with a specific focus on its unique role in the hypothalamus and anterior pituitary. Additionally, the review demonstrates the impact of GnRH on regulating reproductive processes in dairy cows, particularly its involvement in estrous synchronization.

## 2. Structure of GnRH and Its Agonist

The fundamental structure of mammalian GnRH is depicted in Figure 1A. GnRH has two functional terminals. The C terminal (Pro-Gly-NH_2_) is essential for receptor binding, whereas the N-terminal (pGlu-His-Trp-Ser) primarily induces receptor activation, particularly His^2^ and Trp^3^ in sequence [17]. Substituting residues in the N-terminal domain may affect receptor activation by modifying the peptide’s structure (antagonists). In addition, substituting amino acid residues in the C-terminal region is essential for agonist synthesis [2].

After characterizing the decapeptide sequence of GnRH, which is conserved in all mammals [18], the focus shifted to modifying the sequence for producing agonists (Figure 1B) to increase potency and have distinct fertility-enhancing effects. Modifications primarily targeted the C-terminus, increasing agonist effectiveness in inducing ovulation [2]. For example, substituting Alkylamines in the C-terminal instead of the NH2-group enhances the efficacy of agonists in inducing ovulation, as seen in the case of Fertirelin. Likewise, using Alkylamines instead of Gly^10^-NH2-terminus in the C-terminal results in the creation of nonapeptide agonists with improved ovulation-inducing effectiveness, as exemplified by [Pro^9^-ethylamide (NEt)]-GnRH. While the Pro^9^-alkylamine modification resulted in a prolonged duration of action and increased potency, the alkylamine terminus alone did not increase potency [2,8]; furthermore, modifying Gly^6^ in the GnRH sequence enhanced the potency of the agonists. The efficacy of the analog was improved by replacing Gly^6^ with D-Trp^6^ and D-Ser^6^ residues in Treptorelin and Goserelin, respectively. Additionally, the simultaneous alteration of D-Gly^6^ and Pro^9^-ethylamine (NEt), as observed in Nafarelin, Buserelin, and Desloreline, have multiplicative effects on analog potency and efficacy. These modifications also increased the lipophilicity of amino acid substitutions, enhancing drug retention and half-life [19].

These more effective analogs exhibit counterintuitive anti-fertility effects, rapidly recognized and characterized. Agonists with the greatest receptor binding, activation potency, and lower degradation rates demonstrated notable anti-fertility actions. It became advantageous to prevent the first acute release (or “flare effect”) of gonadotropins inherently associated with the agonists by using antagonists with substantial receptor-binding capacity but no receptor activation. Therefore, it was necessary to elucidate the associations between agonist potency, dosage, and treatment period to understand the biological characteristics of GnRH agonists. These characteristics determine whether pro- or anti-fertility effects are associated with treatments with specific peptides [8].

## 3. GnRH Gene Expression in Hypothalamus

**Figure 2:**
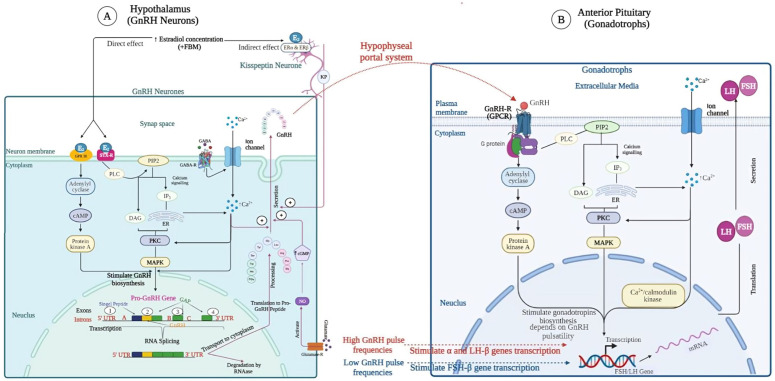
Schematic diagram depicting (**A**) GnRH biosynthesis in hypothalamic GnRH neurons and (**B**) gonadotropins (FSH/LH) synthesis in the anterior pituitary (gonadotrophs). “↑” increase; “1, 2, 3, 4” Exons of primary RNA transcript of GnRH; “A, B, C” Introns primary RNA transcript of GnRH. Created with BioRender.com.

Figure 2A illustrates the biosynthesis process of GnRH in the hypothalamus. In the nucleus of GnRH neurons, the primary RNA transcript of GnRH has three introns and four exons. The first exon (5′ UTR) includes the 5′ untranslated region. In contrast, the second exon encodes the signal peptide GnRH, the enzyme amidation, the precursor processing site, and the initial 11 amino acids of GAP. The third exon encodes the amino acids of GnRH-associated peptide (GAP); the fourth exon includes the 3′ untranslated region (3′ UTR) and the remaining amino acids of GAP. The RNA splicing process produces pro-GnRH mRNA, which is transported to the cytoplasm for translation into the peptide. Post-translational processing, mRNA stability, and transcription rate collectively influence the production of mature GnRH peptides [8,20].

GnRH gene expression is pivotal for regulating reproductive competence. Its complementary DNA, encoding a decapeptide, includes a signal peptide at the amino terminus and a Gly-Lys-Arg sequence followed by GAP at the carboxy terminus [1]. After processing, a 10-kD protein is encoded by the pro-GnRH gene, which contains two peptides—GnRH and GAP—each with 10 and 56 amino acid residues. GnRH is released into the portal vascular system in a pulsatile pattern. While GAP inhibits prolactin secretion in rats, its physiological function in other mammals remains unknown. GnRH initiates neuroendocrine processes that regulate gametogenesis, gonadal steroid production, and pituitary hormone release [21]. The coding region and signal peptide sequence of GnRH genes across species are highly conserved, with two crucial transcription regions in the 5′-side region of the gene with substantial similarity between species [20].

GnRH biosynthesis and secretion regulation involve a complex network of excitatory and inhibitory factors, as illustrated in Figure 2A. This intricate control includes central regulation by distinct subgroups of neurons that connect to GnRH-secreting neurons. Neurotransmitters and neuropeptides, such as Glutamate, Gamma-aminobutyric acid (GABA), Neuropeptide Y (NPY), Leptin, and Gonadotropin-inhibitory hormone (GnIH), have essential functions in this regulatory network. Other intermediates, including protein kinase A (PKA) activators, protein kinase C (PKC), Ca^2+^, and Phospholipase C (PLC), contribute to the modulation of GnRH release. Furthermore, peripheral gonadal steroid feedback mechanisms have been implicated in the overall regulation of GnRH [22,23,24].

## 4. Mechanism of Action of GnRH Analog

To stimulate the production of FSH and LH by pituitary gonadotropic cells (gonadotrophs) in the anterior pituitary, GnRH must directly bind to its receptor, GnRH-R [2,15]. GnRH-R belongs to the G protein-coupled receptor (GPCR) family and is a membrane protein. Upon GnRH binding to the extracellular domain of GnRH-R, a conformational shift occurs, converting the receptor to its active state. Following activation confirmation, the GnRH molecule transmits a signal by interacting with linked G-proteins, mainly through Gq/11-protein-mediated stimulation [21].

Figure 2B illustrates two significant cellular reactions after GnRH binds to its receptor. First, there is a substantial inflow of Ca^2+^ into cells, followed by the activation of calmodulin, an intermediate calcium-binding messenger protein. Second, there is an enhancement in the production of membrane-associated lipid-like diacylglycerols (DAG) as a second messenger, activating the enzyme PKC. PKC, in turn, promotes Mitogen-activated protein kinase (MAPK) cascades in pituitary cells, leading to increased transcription of LH and FSH [25]. Consequently, active calmodulin and activated PKC collaboratively facilitate the release of gonadotropins. Simultaneously, DAG amplifies the activity of Ca^2+^ calmodulin, contributing to the release of gonadotropins [26].

GnRH-R further promotes various signaling pathways in gonadotrophs, including cytoskeletal remodeling and cyclic AMP-dependent protein kinase A (cAMP/PKA) signaling [27]. The cAMP/PKA pathway can induce the biosynthesis and transcription of gonadotropin subunits by incorporating cAMP response elements as promoter subunits. Cytoskeletal remodeling is essential for enhancing the tyrosine phosphorylation state of cytoskeleton-associated proteins, imparting an antiapoptotic effect on neurons and preventing neurodegeneration [8,27,28]. Moreover, the activation of GnRH-R indirectly stimulates PLC, leading to the hydrolysis of membrane phosphoinositide into inositol 1,4,5 trisphosphate (IP3). This IP3 promotes intracellular Ca^2+^ mobilization [8]. GnRH facilitates the entry of Ca^2+^ into gonadotrophs through voltage-operated channels, resulting in an increased cytosolic Ca^2+^ concentration primarily responsible for LH release [26].

Notably, the pulsation of GnRH secretion precisely regulates the transcription of gonadotropin subunit genes: higher pulse frequencies of GnRH lead to increased transcription of α and LH-β. In comparison, lesser GnRH pulse frequencies enhance FSH-β gene transcription [14].

## 5. GnRH Regulation of the Female Estrous Cycle

**Figure 3:**
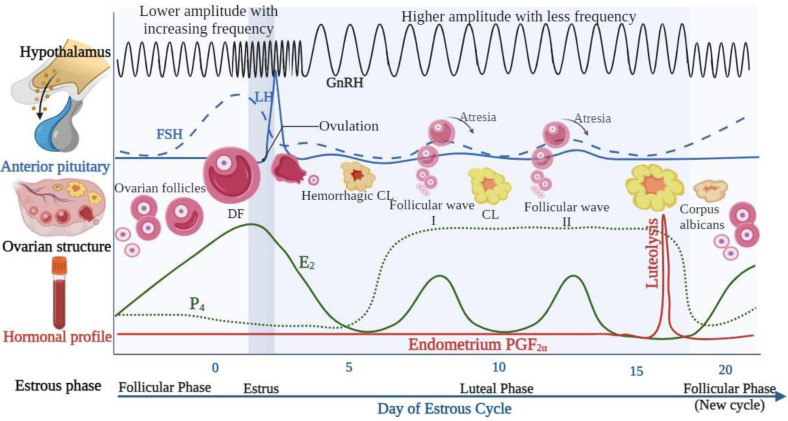
Schematic representation illustrating changes in pulsatile frequencies of GnRH and effects on gonadotropin synthesis in the anterior pituitary (LH, FSH), ovarian structures (pre-ovulatory follicles and CL), and the hormonal profile (E_2_, P_4_, and PGF_2α_) during distinct phases of the estrous cycle (follicular, estrous, and luteal phase). Created with BioRender.com.

The follicular phase (Pro-estrous) begins with corpus luteum (CL) regression (luteolysis) and ends with estrus (Figure 3). Due to luteolysis, progesterone (P_4_) concentrations rapidly decrease, alleviating the negative feedback on the pituitary. Relatively lower P_4_ concentrations lead to a marked increase in the frequency of GnRH pulses that are of lesser amplitude than when the frequency is at a lesser rate, increasing FSH output [29]. FSH and LH coordinate activities on the theca and granulosa cells, producing follicular 17β-estradiol (E_2_). Higher E_2_ concentrations in the blood induce estrous behavior, during which the cow exhibits sexual receptivity and readiness to be mounted [30]. Following the increase in E_2_ concentration, the pre-ovulatory gonadotropin surge, uterine contractions (facilitating sperm transfer), and estrous behavior increase [31]. E_2_ enhances the LH receptor content in granulosa cells, preparing the pre-ovulatory follicle for the gonadotropin surge [16]. The LH pulse frequency is essential for influencing the eventual fate of the dominant follicle (DF) [16]. Ovulation of DF occurs when proteolytic enzymes break down the follicular wall, releasing the oocyte for fertilization. This process typically occurs 10–14 h after estrus, indicating the initiation of the luteal phase of the estrous cycle [16,32].

During the first 3–4 days of the luteal phase, known as metestrus, the follicle that underwent ovulation (corpus hemorrhagic) transforms into the CL. This transformation involves the luteinization of the granulosa and theca cells, producing P_4_ [17,33]. Most ovarian blood flow during this phase goes to the CL to support the P_4_ production, which is essential for establishing and maintaining pregnancy [16]. Various hormones and signals from the anterior pituitary, uterus, ovary, and embryo regulate P_4_ concentrations. P_4_ concentrations remain high during diestrus, and the anterior pituitary releases small concentrations of FSH to stimulate recurrent follicle development. These developing follicles do not undergo ovulation due to insufficient LH pulse frequency [34]. The P_4_-dominant luteal phase generates higher amplitude but less frequent LH pulses, which is inadequate for follicle ovulation due to negative feedback. As CL regresses, P_4_ concentrations decrease during proestrus due to the release of uterine prostaglandin (PGF2α) [34,35].

Throughout the estrous cycle in cattle, generally two to three, sometimes one or four, waves of follicular development occur, each with an emergence, selection, and dominance phase following either atresia or DF ovulation [3,34]. Cattle have follicular waves during prepuberty, estrus, pregnancy, and postpartum anestrus [16].

## 6. Application of GnRH in the Reproduction of Dairy Cattle

### 6.1. Synchronization of the Ovarian Follicular Wave Dynamics and Luteal Phase Support

The growth of ovarian follicles in dairy cattle occurs in cyclical patterns known as follicular waves [36]. The initiation of a follicular wave is distinguished by the enlargement of the follicles to over 4 mm in diameter, which is usually recognized after selecting the DF. Once the follicles reach approximately 8.5 mm, a DF is selected from the group of growing follicles, a process termed follicular deviation. After follicular deviation, the DF continues to grow, whereas subordinate follicles exhibit reduced growth rates and eventually regress in size [37]. Both ovarian follicles and CL are responsive to gonadotropins. GnRH may exert its effects indirectly by releasing LH and FSH or directly impacting reproductive tissues [15].

Previous research has demonstrated that repeated administration of low-dose GnRH stimulates terminal follicle development in cattle. For instance, in one study, dairy cows received 2.5 µg of GnRH at 2-h intervals over 2 days during the early postpartum period, leading to increased concentrations of LH and E_2_. This process restores basal LH secretion during the early postpartum period, initiating the recrudescence of ovarian follicular function [38]. Additionally, during the luteal phase of the estrous cycle, high P_4_ concentrations inhibit E_2_ secretion, resulting in the turnover of anovulatory follicle waves [39]. This occurs because of the negative feedback effect of luteal phase P_4_ concentrations, which reduces pulsatile secretion of LH [40]. Furthermore, studies have indicated that pulsatile administration of GnRH (5 µg at 1-h intervals) during the luteal phase (days 5–11) elevates E_2_ to typical pre-ovulatory peak concentrations within 3 days of administration. These findings support follicular development despite luteal phase P_4_ concentrations, highlighting the significance of LH pulse frequency in regulating terminal antral follicle development [41].

Therefore, delivering GnRH through an appropriate system could effectively regulate follicle selection and dominance by inducing a proestrus LH pulse frequency of approximately one pulse per hour. This regulation can be achieved through pharmacological injections of GnRH agonists, offering an alternative to repetitive hormone administration. These injections trigger a surge of LH and FSH, similar to the natural pre-ovulatory surge [42]. This surge can lead to a decline in plasma E_2_ and inhibin concentrations [39], associated with luteinization, periestrous DF ovulation, and anovulatory DF selection over 7 days [43,44]. The initiation of this entire sequence of events during the estrous cycle upon GnRH administration depends on the ovarian follicular status at the time of injection.

The impact of a single injection of a GnRH agonist (buserelin, 10 μg) on ovarian follicles in cows and heifers was examined when administered between days 11 and 13 of the cycle [45]. While the total count of follicles observed on the ovaries remained unchanged with GnRH treatment, there was a noticeable increase in the number of cloudy follicles across small (3–5 mm), medium (6–9 mm), and large (over 9 mm) follicular classes, accompanied by a decrease in clear follicles compared to the untreated group. The “cloudy follicles” refer to follicles containing flocculent material or with an indistinct basement membrane, resulting in a cloudy appearance of the follicular fluid instead of a clear black appearance during ultrasound examination. This cloudy appearance may be linked to luteinization induction [15]. The changes induced by Buserelin were evident by day 13 for medium and large follicular classes, but they were more pronounced on days 14 and 16. The average count of medium and large follicles, whether clear or cloudy, remained similar on days 18 and 20, suggesting that the effects of treatment on follicular status persisted for 4–6 days. By day 20, a large clear follicle had emerged in each animal injected with Buserelin, becoming the ovulatory follicle [45]. A single clear ovulatory follicle appeared by day 20, indicating a newly selected follicle compared to the untreated group. Essentially, treatment with the GnRH agonist synchronized follicular development, leading to ovulation of the DF present at the time of injection [15,45]. Similar alterations in ovarian follicular dynamics induced by GnRH (buserelin) have corroborated earlier findings [46]. The administration of buserelin may lead to premature luteinization, which could impact the functional integrity of developing follicles’ theca and granulosa layers, potentially affecting their capacity to produce E_2_ [46].

Furthermore, studies have shown that GnRH agonist (buserelin) injection reduces the variability in plasma E_2_ concentrations within cows [45]. Similarly, administering a less potent GnRH agonist (fertirelin) between days 11 and 13 of the estrous cycle resulted in a dual response in serum E_2_ levels: an acute increase occurred between 4 and 6 h post-administration, followed by lower E_2_ concentrations over the subsequent 7-day period [47]. These findings further emphasize the role of GnRH agonists in regulating follicular development and function during the luteal phase of the estrous cycle.

Several studies have shown that administering GnRH or GnRH agonists around the LH surge time can enhance the induced pre-ovulatory LH surge, potentially affecting oocyte maturation [48,49]. The LH surge, essential for oocyte maturation, does not differ significantly in timing, regardless of conception status post-AI [50]. Injecting a GnRH agonist during this surge can increase LH concentrations and potentially influence oocyte maturation. For instance, in heifers treated with a GnRH agonist after PGF_2α_ injection, oocyte maturation was accelerated by 65 h, accompanied by a notably heightened LH surge compared to untreated heifers [51]. These findings emphasize the importance of timing GnRH injections with the natural LH surge to optimize oocyte maturation and ovulation efficiency, crucial factors in enhancing reproductive performance in dairy cattle.

**Figure 4:**
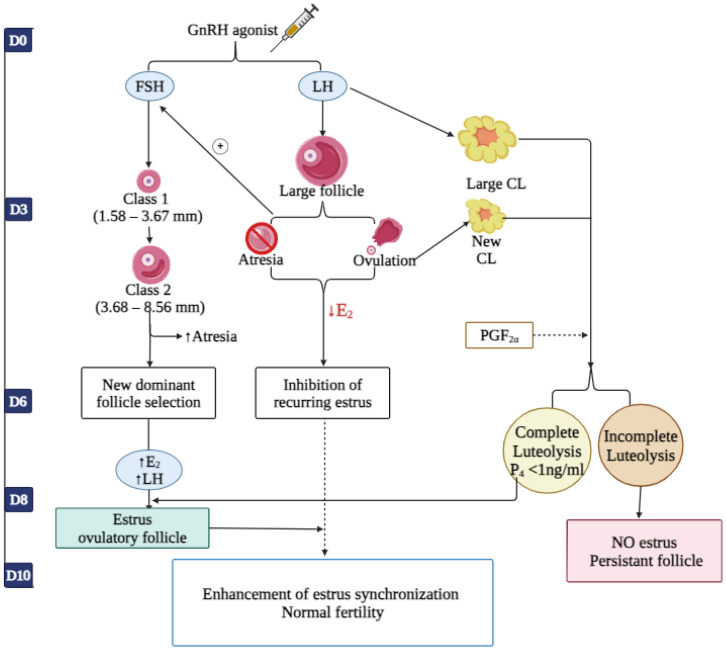
The illustration depicts the potential impact of administering a GnRH agonist in a 10-day treatment regimen on ovarian follicular dynamics and luteal cell function, aiming to improve the precision of estrus in cattle. Adapted from [52]. Created with BioRender.com.

In conjunction with the estrous synchronization of dairy cattle using GnRH and its agonists, illustrated in Figure 4, the administration of a GnRH agonist within a 10-day program has shown positive effects on ovarian follicle dynamics and CL function, enhancing the synchronization of the estrous cycle stage without compromising fertility outcomes [52]. The injection of GnRH agonist on day 0 (the day of GnRH administration) in cows, for which the estrous cycle stage is unknown, induces an increase in circulating FSH and LH. These hormones influence ovarian follicles and CL development [53]. Depending on the developmental stage and as a response to GnRH-induced LH, the largest follicle disappears due to ovulation and the development of a CL or atresia. Consequently, E_2_ concentration decreases in both cases, preventing recurring estrus in 6 days relative to the treatment time with GnRH [15]. When a CL is present at GnRH administration, the GnRH-induced LH release leads to the development of larger luteal cells (LLC). At the same time, FSH stimulates atresia of small (1.58–3.67 mm) or medium (3.68–8.56 mm) follicles. There is a greater rate of atresia in medium follicles, preventing further development into larger ones.

The induced follicular wave dynamics selected a new DF within 3 to 4 days post-treatment with GnRH. On day 7, complete luteolysis is generally caused by PGF_2α_ administration. The E_2_ concentration subsequently increases, and behavioral estrus occurs, inducing a pre-ovulatory LH surge leading to ovulation from the DF [54]. During the interval between days 7 to 10, the percentage of cows expressing estrus and the synchrony during estrous onset are enhanced, resulting in typical fertility outcomes in GnRH-treated cows. However, a small percentage of cows do not express behavioral estrus due to incomplete PGF_2α_-induced luteolysis, resulting in the development of the DF into what has been termed a persistent follicle [54,55].

The concentration of P_4_ and variations in the types of steroidogenic luteal cell proportions (large and small luteal cells: LLC and SLC) are indicators of the effects of GnRH agonist administration on the follicular dynamics and the function and morphology of CL [55]. It has been reported that the administration of GnRH agonist (Buserelin) can affect the function and morphology of CL. Even without changes in P_4_ concentration, administering GnRH stimulates morphological alterations of CL and enhances the number of large luteal cells (LLCs) and size of the CL within 6 days [45,47,55]. Moreover, the administration of GnRH agonist Cystorelin to repeat breeder cows at the time of estrus (12 h after estrus onset) increases the number of LLCs in the CL, which leads to an increase in the concentrations of P_4_ earlier after ovulation and maintains it higher up to 40 days during pregnancy [56]. There have been conflicting reports about the effects of the GnRH agonist on the concentration of P_4_ in cows. Some studies have reported positive effects [45,57,58], while others have reported adverse effects [59]. When cows are treated with GnRH agonist (Buserelin), there is an increase in the number of LLCs due to the release of LH [60]. As PGF_2α_ receptors are mainly present in the plasma membranes of LLCs, the treatment with GnRH may result in a greater probability of luteolysis [52]. It has been observed that the decrease in P_4_ concentration within 24 h following PGF_2α_-induced luteolysis indicates that both the CL present at the time of GnRH administration and the induced CL respond similarly to exogenous PGF_2α_. Therefore, the GnRH-induced CL and naturally developing CL are equally affected by PGF_2α_-induced luteolysis [52]. The inconsistencies in the results of GnRH responses on P_4_ concentration may be associated with the timing of GnRH administration during the estrous cycle, stimulation of accessory CL, or the type of GnRH analog used for treatments.

### 6.2. Hormonal Control of the Timing of Behavioral Estrus among Cows Using GnRH and PGF_2α_

The aim of the GnRH-PGF_2α_ treatments is to synchronize the stages of the estrous cycle (follicular phase and luteal phase) in dairy cows using two hormonal treatments: (1) modifying/imitating the function of CL and (2) controlling the timing of follicular development and ovulation [15,16].

Using exogenous GnRH analogs with PGF_2α_ is a hormonal manipulation method to regulate the estrous cycle by influencing ovarian structures (follicles, CL), thus affecting hormonal profiles [32]. Exogenous GnRH functions similarly to endogenous GnRH during the natural estrous cycle without any treatments. The surge release of GnRH is induced by the effects of E_2_ at the pulse center of the hypothalamus during the follicular phases of estrous cycles in the absence of exogenous treatments. This surge in GnRH production prevents the growth of existing follicles, leading to the emergence of new follicular waves. Approximately 1 or 2 days following the injection of exogenous GnRH analog, a new follicular wave is initiated [61,62].

Consequently, all treated cows should be at the same stage of follicular dynamics when PGF_2α_ is administered 7 days following the GnRH analog administration. The administration of PGF_2α_ induces regression of the CL, and the development of DF occurs within 2 to 3 days following treatment with the analog. Additionally, GnRH induces the DF to luteinize, potentially stimulating the onset of estrous cycles in anestrous cows [61].

Estrous synchronization using GnRH–PGF_2α_-based treatment regimens is widely utilized in dairy herds due to its effectiveness, eliminating the need for estrous monitoring and allowing for precise insemination timing relative to ovulation. Consequently, there were comparable pregnancy rates per insemination (P/AI) compared to conventional reproductive management programs [63]. Various modifications have been made to GnRH–PGF_2α_-based treatment regimens for estrous synchronization. A common aspect of these treatment regimens is the 7-day interval between GnRH and PGF_2α_ administration. However, the differences lie in how animals are managed for estrous detection and artificial insemination (AI) [32].

Among the GnRH–PGF_2α_ treatment regimens aimed at synchronizing the estrous cycle in cows, the Ovsynch or GPG (GnRH–PGF_2α_–GnRH) treatment regimen is the prevalent “fixed-time” insemination regimen. This regimen involves an initial GnRH–PGF_2α_ treatment, spaced 7 days apart, followed by a second GnRH treatment 2 days after the PGF_2α_ treatment [64]. It is administered at random stages of the estrous cycle and aims to equalize follicle development in the ovaries, induce ovulation, and facilitate AI [65]. In cows with a functional DF, the first GnRH treatment is intended to stimulate ovulation of the developed ovarian follicles, leading to CL formation. In cows without a functional DF, the first GnRH treatment promotes the development of a new follicular wave, culminating in the formation of a DF by the end of the Ovsynch regimen [65,66]. Seven days later, the administration of PGF_2α_ induces luteolysis in cows that have been treated 7 days earlier with GnRH and allows for the ongoing development of the DF of the next wave [67]. As the dominant follicle grows, circulating E_2_ levels increase, and cows exhibit estrus approximately 48 h after PGF_2α_ treatment. Subsequently, 48 h after PGF_2α_ administration, the second GnRH injection induces LH surge, synchronizing ovulation and allowing for proper timing of AI before ovulation [66]. However, the precise timing of insemination varies depending on the specific treatment regimen, ranging from 8 to 20 h [47], 16 to 20 h [48], or even between 13 to 16 h [49] after the second GnRH treatment, respectively. With the Ovsynch treatment, dairy cattle can achieve a moderate pregnancy rate of around 40% without the requirement for estrous detection, which is considered an excellent outcome [32,68].

Consequently, various modifications to the Ovsynch regimen, such as Cosynch, Presynch, Select-synch, and Heat-synch, have been developed and utilized in alternative treatment regimens for specific scenarios. Table 1 provides a detailed overview of studies evaluating and comparing the reproductive efficacy of dairy cattle subjected to Ovsynch and modified treatment regimens.

**Figure 5:**
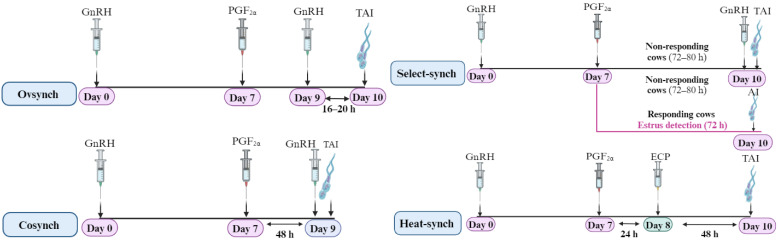
The schematic diagram depicts various fundamental estrous synchronization protocols (Ovsynch protocol and its modifications). Adapted and modified from [63,77]. Created with BioRender.com.

For all modified treatment regimens, depicted in Figure 5, the crucial aspect retained from the Ovsynch regimen is the unaltered 7-day interval between the first GnRH injection and the subsequent PGF_2α_ injection. One such regimen, Co-synch, involves AI following the second GnRH injection administered simultaneously [69]. In the Heat-synch regimen, estradiol cypionate is administered instead of the second GnRH injection [70]. Presynch refers to attempting to synchronize the stage of follicle development patterns before the first GnRH administration. This can be achieved either by administering two injections of PGF_2α_ or through a combination of GnRH and PGF_2α_ injections. These methods are often employed before initiating various treatment regimens for TAI [71]. Another way to enhance fertility in dairy herds and reproductive efficiency is through resynchronization approaches, intending to reduce the interval to re-insemination and maximize the P/AI [72].

**Figure 6:**
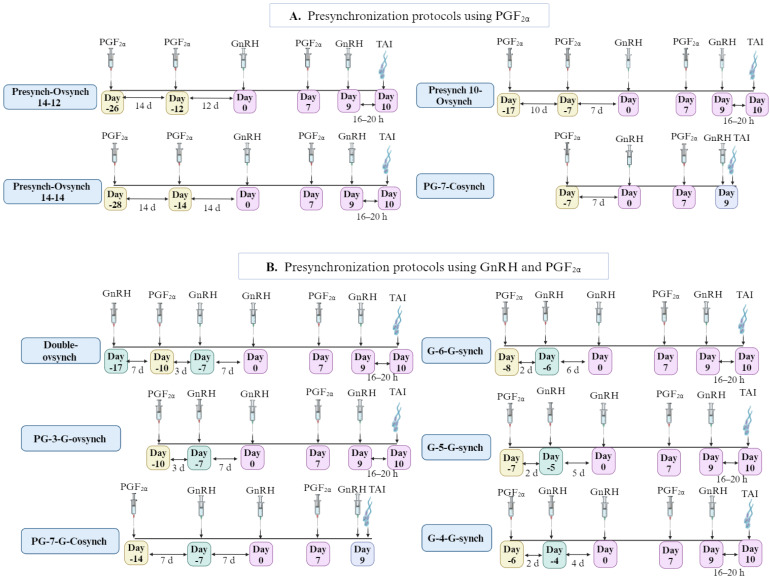
The schematic diagram depicts various fundamental presynchronization protocols using PGF_2α_ (**A**) or combining GnRH and PGF_2α_ (**B**), encompassing the Ovsynch protocol and its modifications. Adapted and modified from [63,77]. Created with BioRender.com.

Presynch treatment regimens (Figure 6A) involve two injections of PGF_2α_ at 12 to 14-day intervals before initiating the Ovsynch treatment regimen [73,74,75,76]. This prostaglandin treatment leads to cows in the same stage of the estrous cycle at the time of administration of the estrous synchronization treatment regimen. Results in Table 2 indicate a markedly enhanced conception rate in herds where presynchronization treatments were implemented, especially in multiparous dairy cows [73]. The Presynch treatment regimen is widely used to enhance herd reproductive efficiency; however, it has certain limitations. One concern is the initiation of the Presynch regimen before the end of the voluntary waiting period (VWP), which can cause adverse effects on cows’ reproductive health. Administering the regimen during VWP, before uterine involution is complete, induces luteolysis in cows with functional CL, consequently reducing the conception rate. Therefore, initiating the Presynch treatment regimen is recommended only after the VWP is completed [32,68].

**Table 1:** The effect of Ovsynch treatment regimen administration and its modifications on the reproductive performance of Holstein dairy cows.

Ref	Animal and Physiological Stage	Treatment Regimen	Modifications	Fertility Outcomes						Summary and Limitations
				Days of TAI	Inseminated %	Ovulation %	Conception %	Pregnancy %	Embryo Loss %	
[78]	Ovular cows (n = 117)	Ovsynch	-	-	-	97.0	32.0	32.0	14.0	OV effectively synchronized ovulation in both groups but did not improve the reproductive performance of anovular cows.
	Anovular cows (n = 33)			-	-	94.0	9.0	9.0	0.0	
[79]	Lactating dairy cows (58–78 DIM)	Ovsynch (n = 115)	-	68 ± 1.1	100	-	35.6	35.6	-	Pregnancy and Conception rates tended to be greater after OV because of poor expression of estrus.
		Select-synch (n = 112)	AI 10–14 h after estrous detection	73 ± 1.0	68.2	-	41.1	26.8	-	
[64]	Primi- and multiparous lactating dairy cows (50 d postpartum)	Ovsynch (n = 167)	-	54	-	-	-	37.0 (d 60) 53.0 (d 100)	-	OV allowed effective management of AI without the need for estrous detection.
		ED (n = 166)	Estrus detected using the a.m.–p.m. rule.	83	-	-	-	5.0 (d 60) 35.0 (d 100)	-	
[80]	Heifers (13 to 23 months)	Ovsynch (n = 77)	-	9 ± 0	-	-	-	74.4	-	Cows in the OV group that were >76 d postpartum had a greater pregnancy rate per AI than cows that were 60 to 75 d postpartum.
		ED (n = 78)	-	13 ± 11	-	-	-	35.1	-	
	Multiparous dairy cows (60–289 d postpartum).	Ovsynch (n = 156)	-	9 ± 0	-	-	-	37.8	-	
		ED (n = 154)	-	13 ± 11	-	-	-	38.9	-	
[81]	Holstein dairy cows (n = 40) with normal reproduction at 70–110 d postpartum.	Ovsynch full dose (n = 20)	10.5 µg buserelin acetate	-	-	-	85.0	50.0	-	5.25 µg buserelin is as effective as the full dose (10.5 µg) in the OV protocol of lactating dairy cows.
		Ovsynch half dose (n = 20)	5.25 µg buserelin acetate	-	-	-	90.0	40.0	-	
[82]	Repeat breeding crossbred cows	Ovsynch (n = 6)	-				50.0	-	-	The incidence of accessory CL formation in treated cows after GnRH treatment on day 6 of the estrous cycle was high.
		Control (n = 6)	Not treated	-	-	-	0.0	-	-	
[83]	Primiparous and multiparous lactating dairy cows (n = 161) with a mature CL and a follicle with >10 mm.	Shortened Ovsynch (n = 22)	-	-	-	-	36.4	33.3	-	If a CL can be detected during reproductive examination in which the heat detection rate is poor, a shortened OV can be recommended. Shortened OV reduced the pregnancy rates for cows that ovulated late compared to the control group.
		Control (n = 73)	One injection of PGF_2α_	-	-	-	41.1	56.3	-	
[84]	Nonpregnant cows from three herds Eligible for reinsemination between 26–29 d after the 1st AI.	Shortened Ovsynch (n = 160)	-	31 ± 1	-	-	23.3	85.6	-	Conception and overall pregnancy rates did not differ significantly between groups. Shortened OV significantly reduced days to TAI.
		Control (n = 189)	Not treated	55 ± 1	-	-	22.8	75.9	-	
[85]	German Holsteins with ovarian cysts. On days 55 to 60 postpartum.	Ovsynch (n = 65)	M-OV: 1st GnRH + 1st PGF_2α_ (d 0), 2nd PGF_2α_ (d 14) and 2nd GnRH (d 16)	74.8 ± 1.5	-	-	42.9	83.1	-	OV can be used to treat ovarian cysts. The M-OV protocol led to a better cure rate and reproductive performance than the OV protocol.
		Modified Ovsynch (n = 65)		69.9 ± 1.5	-	-	27.3	60.0	-	
[86]	Holstein crossbred cows from 5 herds.	Ovsynch (n = 851)	M-OV: Ovsynch with an additional PGF_2α_ (d 8)	-	-	-	42.0	-	-	M-OV protocol increased conception rates and decreased P_4_ at insemination day compared with cows receiving OV protocol.
		Modified Ovsynch (n = 852)		-	-	-	49.0	-	-	
[87]	Holstein dairy cows with CL and at least one follicle >10 mm in size	Ovsynch (n = 161)	M-OV: Injecting hCG instead of 1st GnRH in the Ovsynch	-	59.6	-	-	48.5	0.05	Administration hCG is not a suitable replacement for the 1st GnRH of OV due to its adverse impact.
		Modified Ovsynch (n = 210)		-	65.7	-	-	37.6	0.05	
[88]	Lactating Holstein cows with normal postpartum intervals to the first service	Ovsynch (n = 31)	-	84 ± 10	-	-	29.0	93.5	-	OV produced higher fertility, superior pregnancy rates and fewer days to FAI than AI at estrus detection in cows inseminated in early postpartum ≤ 100 DIM.
		Select-synch (n = 42)	Monitored for estrus signs for 5 d and AI	117 ± 7	-	-	26.2	85.7	-	
[89]	Nulliparous heifers and lactating cows Treated females exhibited extended intervals between AI (27 to 53 d since their previous AI).	Ovsynch (n = 224)	-	-	82.0	-	-	37.0	-	Pregnancy outcomes were similar between the OV and Heatsynch protocols. AI after detecting estrus before the scheduled TAI resulted in shorter days to conception and tended to increase conception rates, especially with the Heatsynch protocol.
		Heat-synch (n = 230)	-	-	62.0	-	-	29.0	-	
[69]	Holstein multiparous cows (n = 54)	Cosynch	-	-	-	50.0	-	41.0	-	Cosynch protocol is more effective in heifers than multiparous cows. Ovulations that occurred before AI could be the reason for the low conception rate of OV.
	Heifers (n = 53).		-	-	-	35.0	-	51.0	-	

AI: artificial insemination, CL: corpus luteum, d: day, DIM: days in milk, ED: estrus detection, GnRH: gonadotropin-releasing hormone, h: hour, hCG: human chorionic gonadotropin, mo: month, n: number, OV: Ovsynch, P_4_: progesterone, PGF_2α_: prostaglandin, TAI: fixed-time artificial insemination.

**Table 2:** Estrous presynchronization treatment regimens using PGF_2α_ in Holstein dairy cows.

Ref	Animal and Physiological Stage	Treatment Regimen	Modifications	Fertility Outcomes						Summary and Limitations
				Days of TAI	Inseminated %	Ovulation %	Conception %	Pregnancy %	Embryo Loss %	
[76]	Primiparous (P) and multiparous (M) lactating Holstein cows at ~60 to 70 DIM until dry-off.	Presynch-14–12- OV (n = 1566)	-	-	-	-	-	40.5 (P) 31.2 (M)	6.4 (P) 6.3 (M)	Extending the duration from 12 to 14 d apart from Presynch to OV decreased ovulatory response but did not reduce the fertility of cows that received TAI.
		Presynch-14–14- OV (n = 1599)	-	-	-	-	-	36.5 (P) 36.7 (M)	5.2 (P) 7.1 (M)	
[90]	Holstein dairy cows (60 DIM). Treatment at random stages of the estrous cycle.	Ovsynch (n = 134)	-	-	-	69.6	37.3	-	-	Presynchronization protocol increases the PR/AI of lactating dairy cows receiving TAI compared with OV.
		Presynch (n = 135)	Ovsynch but with the addition of 2 PGF_2α_	-	-	81.1	49.6	-	-	
[91]	Lactating dairy cows at 24 to 44 DIM All cows received Presynch- 14- 12- OV and then different Ovsynch.	G48-AI48 (n = 224)	GnRH + TAI at 48 h after PGF_2α_	-	-	-	-	22.8	5.9	GnRH and AI administration at 72 h after PGF_2α_ in the Presynch 14 (G72-AI72 group) enhances pregnancy rates and reduces pregnancy loss compared to other groups.
		G48-AI72 (n = 221)	GnRH at 48 h + TAI at 72 h after PGF_2α_	-	-	-	-	23.5	13.3	
		G72-AI72 (n = 220)	GnRH + TAI at 72 h after PGF_2α_	-	-	-	-	31.4	1.6	
[92]	Lactating cows from 2 herds (59 to 79 DIM)	Presynch-14–12-OV (n = 318)	-	-	68.0	-	-	46.8	-	Presynch administration before OV protocol is recommended due to the increasing pregnancy rate in dairy cows.
		Ovsynch (n = 312)	-	-	73.0	-	-	37.5	-	
[93]	Lactating Holstein–Friesian dairy cows treated at 30–35 d postpartum	Presynch-14–12- OV (n = 100)	-	64.2 ± 4.2	-	-	61.0	-	-	Using PG + P-OV significantly reduces days to conception and NSC and improves P/AI.
		PG + Presynch-14–12- OV (n = 41)	+ PGF_2α_ 15 d before applying Presynch.	60.4 ± 3.7	-	-	87.8	-	-	
		Control (n = 100)	Not treated	79.1 ± 4.8	-	-	46.0	-	-	
[94]	Lactating Holstein cow treated at 30 days postpartum.	Presynch-14–12- OV (n = 446)	Primiparous		-	-	31.8	-	-	Primiparous cows responded more favorably to Presynch administration than multiparous cows by increasing conception rates and incidence of normal inter-estrus interval.
		Presynch-14–12- OV (n = 726)	Multiparous				26.3			

(%) percentage, AI: artificial insemination, d: day, DIM: days in milk, GnRH: gonadotropin-releasing hormone, n: number, NSC: number of services per conception, OV: Ovsynch, P/AI: pregnant rate per insemination, PG: prostaglandin, PGF_2α_: prostaglandin, P-OV: Presynch-Ovsynch.

The presynchronization treatment regimen for synchronizing the estrous cycle among cows may involve the administration of GnRH (Figure 6B). This is performed to maximize this hormone’s ovulation induction effects and enhance the percentage of P/AI when using TAI. Incorporating GnRH before implementing estrous detection-based presynchronization treatment regimens in lactating dairy cows can improve the reproductive efficiency of anovulatory cows by inducing ovulation before initiating a PGF_2α_-Presynch treatment regimen [95]. However, using GnRH alone before initiating the Presynch procedure reduces behavioral estrous expression, which is undesirable in TAI breeding programs where behavioral estrous expression is correlated with fertility [96]. Combining GnRH and PGF_2α_ (Table 3) enhanced presynchronization outcomes.

There have been evaluations of a Presynch procedure using PGF_2α,_ and GnRH administered 2 days apart at different intervals, such as 4-, 5-, or 6-day intervals, before implementing the Ovsynch treatment regimen [97]. It was concluded that imposing the PGF_2α_-GnRH Presynch treatment regimen at a 6-day interval before initiating the Ovsynch treatment regimen led to increased fertility, a higher percentage of cows experiencing ovulations, and a luteolytic response. Lactating dairy cows administered a PGF_2α_-3-GnRH Presynch treatment regimen at a 7-day interval before initiating the Ovsynch treatment regimen had greater ovulation rates, improved luteal function, and increased pregnancy rates compared to cows subjected to presynchronization using PGF_2α_-PGF_2α_ administration 14 days apart [74].

Another evaluation involved a Presynch treatment regimen injecting PGF_2α_-GnRH on the same day, 7 days before the onset of an Ovsynch treatment regimen, instead of using the PGF2α-3-GnRH treatment regimen with administration 3 days apart [98]. This alternative Presynch treatment regimen resulted in a higher pregnancy rate per the first AI, an increase in the percentage of cows with ovulations after the first GnRH of the Ovsynch treatment regimen, and a greater concentration of P_4_ at the time of Ovsynch [98].

Furthermore, consistent results have been reported in several other studies [91,99,100] employing a Cosynch treatment regimen in combination with a PGF_2α_-based or PGF_2α_-GnRH-based presynchronization program. This combined Cosynch treatment regimen demonstrated efficacy in synchronizing the estrous cycle among cows [32].

**Table 3:** Estrous presynchronization using the combination of GnRH and PGF_2α_ in Holstein dairy cows.

Ref	Animal and Physiological Stage	Treatment Regimen	Modifications	Fertility Outcomes					Summary and Limitations
				Synchronization %	Ovulation %	Conception %	Pregnancy %	Embryo loss %	
[95]	Primiparous lactating dairy cows (at 60 ± 3 DIM) during heat stress.	Presynch-Cosynch (n = 123)	-	-	50.6	-	32.1	8.2	Administration of GnRH before presynchronization increases P_4_ concentration (3.6 ± 0.3 ng/mL) compared to control (2.7 ± 0.4 ng/mL), improving fertility parameters under heat stress conditions.
		GnRH- Presynch-Cosynch (n = 102)	+ GnRH before applying presynch	-	15.2	-	31.8	6.9	
[72]	Lactating Holstein cows at non-pregnancy diagnosis (d 0).	PG7-Cosynch (n = 967) + TAI	-	-	-	17.2	11.5	23.5	PG7-G7-Cosynch is an effective method to resynchronize cows, resulting in doubled P/AI.
		PG7-G7-Cosynch (n = 962) + TAI	PGF_2α_ on d0 and GnRH on d 7	-	-	28.0	21.2	16.4	
[74]	Postpartum lactating Holstein cows between 36 to 50 d DIM.	PG-3-G (n = 105)	-	-	80.0	-	40.0	7.5	The PG-3-G regimen improves ovulation rate and luteal function 7 d before OV, increasing follicular synchrony and P/AI in lactating dairy cows.
		Presynch-10 (n = 105)	-	-	53.3	-	33.3	8.6	
[97]	Lactating Holstein dairy cows (n = 137) before 1st service between 62–70 DIM	G-4-G (n = 33)	-	87.9	56.0	-	24.0	-	G-6-G regimen before applying OV increases ovulatory and luteolytic response OV compared to other regimens
		G-5-G (n = 31)	-	62.9	66.7	-	34.0	-	
		G-6-G (n = 32)	-	92.7	84.6	-	50.0	-	
		Ovsynch (n = 34)	-	77.1	53.8	-	27.0	-	
[98]	Lactating dairy cows at 58 to 64 DIM (first service) and cows diagnosed as not pregnant 39 days after the previous AI (2nd service).	G-6-G (n = 116)	-	-	67.0	-	57.0 (d 35) 54.0 (d 49)	-	However, the P/AI were similar between groups on d 35 and 49; the ovulation rate increased after G-6-G application due to increasing P_4_ concentrations at the time of PGF_2α_ of OV (5.75 ng/mL vs. 4.64 ng/mL).
		PG + G (n = 121)	PGF_2α_ +GnRH 7d before OV.	-	68.0	-	50.0 (d 35) 47.0 (d 49)	-	
[101]	Noncycling lactating Holstein cows at 42 ± 3 DIM.	Double Ovsynch (n = 100)	-	-	98.0	-	-	-	Pre-synchronization using double OV induced ovulation in noncycling cows and appeared to increase synchronization features during the OV protocol.
		Presynch-14–12-Ovsynch (n = 93)	-	-	93.5	-	-	-	
[102]	Lactating Holstein cows (primiparous: P, multiparous: M) at 42 ± 3 DIM	D-OV (n = 157)	-	-	71.8	-	65.2 (P) 37.5 (M)	-	The D-OV regimen increases P/AI only in primiparous and not in multiparous cows. The fertility in primiparous cows compared to P-OV is due to the induction of ovulation in noncycling cows and the improved synchronization of cycling cows.
		Presynch-14–12-Ovsynch (n = 180)	-	-	66.7	-	45.2 (P) 39.3 (M)	-	
[103]	Primiparous (P) and multiparous (M) lactating dairy cows at 45 ± 3 DIM for the presynch group and 54 ± 3 DIM for the double Ovsynch group.	Double-Ovsynch (n = 837)	-	-	-	-	52.5 (P) 40.3 (M)	-	D-OV improved fertility in dairy cows compared to the Presynch regimen, particularly benefiting primiparous cows. D-OV could be a beneficial reproductive management regimen for synchronizing the first service in dairy herds.
		Presynch-14–12-Ovsynch (n = 850)	-	-	-	-	42.3 (P) 34.3 (M)	-	
[104]	Multiparous lactating Holstein cows during the heat-stress season	Double-Ovsynch (n = 486)	-	26.6	-	-	23.2	6.1	D-OV significantly increases the synchronization rate and P/AI in summer. Also, it increases the mean diameter of the ovulatory follicle at TAI by (0.5 mm) D-OV treatment regimen yields optimal reproductive performance in heat-stressed dairy cows.
		Presynch-14-GnRH-Ovsynch (n = 453)	Additional GnRH 2 d after applying presynch	21.4	-	-	16.7	6.6	
		Presynch-14–14-Ovsynch (n = 435)	-	17.2	-	-	12.4	7.4	
[105]	Lactating Holstein cows with VWP 60 ± 3 d: n = 458 and 88 ± 3 d: n = 462.	Double-Ovsynch (D-OV60, n = 458)	-	-	-	-	43.3	5.9	D-OV administration on d 60 and 88 of VWP, P/AI at 39 ± 3 days post-AI was similar among treatment groups.
		Double-Ovsynch (D-OV88, n = 462)	-	-	-	-	45.5	7.1	
[106]	Lactating primiparous and multiparous Holstein and Jersey crossbred	Double-Ovsynch (100, n = 24)	Using 100 or 200 μg of GnRH	-	-	-	-	-	D-OV with 200 μg of GnRH increased LH secretion instead of a 100 μg dose of GnRH, either in a high or low P_4_ concentration.
		Double-Ovsynch (200, n = 22)							
[107]	Lactating primiparous cows (n = 165) between 60 and 172 d postpartum	Double-Ovsynch (n = 81)	-	-	-	-	72.8	-	D-OV regimen increases the pregnancy rates in postpartum primiparous cows compared to the OV regimen.
		Ovsynch (n = 84)	-	-	-	-	29.8	-	
[108]	Lactating Holstein cows at 42 DIM (41 ± 0.1 d). During the year’s warm (W) and cool (C)seasons.	PG-3-G (n = 1286)	-	-	-	-	35.9 (W) 46.8 (C)	-	During the summer, PG-3-G enhanced P/AI compared to Presynch-10. However, no significant difference in P/AI between treatments during cold weather.
		Presynch-10 (n = 1247)	-	-	-	-	26.7 (W) 44.3 (C)	-	

AI: artificial insemination, d: day, DIM: days in milk, D-OV: Double-Ovsynch, GnRH: gonadotropin-releasing hormone, h: hour, LH: luteinizing hormone, n: number, OV: Ovsynch, P/AI: pregnant rate per insemination, P_4_: progesterone, PGF_2α_: prostaglandin, TAI: fixed-time artificial insemination, VWP: voluntary waiting period.

## 7. Conclusions

This review article emphasizes the essential functions of hypothalamic GnRH in regulating reproductive activity. It focuses on how GnRH modulates the production and release of pituitary gonadotropins, which affect steroidogenesis and gametogenesis. Several GnRH-based products, including agonists, have been produced to improve the reproductive efficiency of livestock. This study emphasizes the beneficial effects of GnRH and its agonists in synchronizing the estrous cycle of dairy cows, including synchronizing the dynamics of ovarian follicular waves and providing support throughout the luteal phase. GnRH also has an essential role in controlling the timing of behavioral estrus in cows. As a result, multiple treatment regimens have been developed to synchronize the estrus in livestock. Based on the results of several studies discussed in this review article, it has been found that GnRH-PGF_2α_ regimens are the most efficient approach for synchronizing estrus. This is attributed to eliminating the requirement for estrous detection and the improved control over insemination timing relative to ovulation, leading to an overall enhancement in reproductive efficiency. Despite there being several competing treatment regimens based on GnRH-PGF_2α_, it is challenging to identify the most effective protocol due to the inconsistent outcomes shown in investigations assessing these regimens. Therefore, continuing research and improvement in this field can potentially enhance reproductive efficiency in dairy herds.

## Data Availability

Data sharing is not applicable.
